# Continuous veno-venous hemofiltration in patients with metformin associated lactic acidosis

**DOI:** 10.1186/2197-425X-3-S1-A464

**Published:** 2015-10-01

**Authors:** C Bethlehem, PHJ van der Voort

**Affiliations:** Onze Lieve Vrouwe Gasthuis, Intensive Care, Amsterdam, the Netherlands; TIAS School for Business and Society, Tilburg, the Netherlands

## Introduction

Metformin is frequently used by type II diabetics. A rare complication of its use is metformin associated lactic acidosis (MALA). This condition is often accompanied by extreme metabolic acidosis and hemodynamic instability for which ICU treatment is mandatory. Guidelines advice intermittent hemodialysis for correction of metabolic acidosis and to clear metformin. However, haemodynamic instability may prefer continuous veno-venous hemofiltration (CVVH). We evaluated the effect of CVVH in critically ill patients admitted with MALA.

## Methods

We performed a retrospective single centre cohort study in a 20-bed mixed ICU. All consecutive patients admitted with clinical diagnosis of metformin intoxication between January 2010 and December 2014 were included. Data were extracted from the local Patient Data Management System and analysed with SPSS 21.0. Data are presented as median and IQR or mean ± SD.

## Results

10 patients were identified. 80% were male, median age was 68 years (IQR 65-77). Apache II was 30.5 (IQR 27-34), corresponding with a predicted mortality of 65% (IQR 39-83). The first measured serum metformin level (N = 6) was 32 (IQR 14-50). Nine cases were due to chronic accumulation of metformin and one was an acute intoxication. Metformin accumulation could be explained by renal impairment caused by dehydration (N = 4), infection (N = 3) or medication (N = 2). Mean pH at admission was 7.08 (SD ± 0.136), highest measured lactate was 13.95 mmol/l (IQR 10-21). Continuous veno-venous hemofiltration was initiated in 8 out of 10 patients, the other 2 patients had a pH above 7.20. CVVH was administered in high volume post-dilution mode with citrate anticoagulation. Dosage of CVVH was 54.89 ml/kg/hour (IQR 50.8-61.1). Time to normalisation of pH was 557.5 minutes (IQR 285-840), median duration of CVVH was 35.67 hours (IQR 17-75.33). In three patients the serum level was repeatedly measured and showed a decline. A mean arterial pressure of 60 mmHg was achieved with a maximum concentration of 0.08 mcg/kg/min norepinephrine (IQR 0-0.29) and 5.6 mcg/kg/min dopamine (IQR 2.2-9.8). The two oldest patients died, one because of termination of renal replacement therapy in end-stage kidney disease due to tubulointerstitial nephritis (79 years), the second because of refractory septic shock (85 years).

## Conclusions

CVVH was effective in restoring acid base disorders and haemodynamics in patients with MALA and hypotension. It appears to be a safe alternative to intermittent hemodialysis.
